# Assessment and rehabilitation of neglect using virtual reality: a systematic review

**DOI:** 10.3389/fnbeh.2015.00226

**Published:** 2015-08-25

**Authors:** Elisa Pedroli, Silvia Serino, Pietro Cipresso, Federica Pallavicini, Giuseppe Riva

**Affiliations:** ^1^Applied Technology for Neuro-Psychology Lab, IRCCS Istituto Auxologico ItalianoMilan, Italy; ^2^Department of Psycholgy, Università Cattolica del Sacro CuoreMilan, Italy

**Keywords:** virtual reality, USN, assessment, rehabilitation, PRISMA

## Abstract

After experiencing a stroke in the right hemisphere, almost 50% of patients showed Unilateral Spatial Neglect (USN). In recent decades, Virtual Reality (VR) has been used as an effective tool both for the assessment and rehabilitation of USN. Indeed, this advanced technology allows post-stroke patients to interact with ecological and engaging environments similar to real ones, but in a safe and controlled way. To provide an overview of the most recent VR applications for the assessment and rehabilitation of USN, a systematic review has been carried out. Since 2010, 13 studies have proposed and tested innovative VR tools for USN. After a wide description of the selected studies, we discuss the main features of these VR tools in order to provide crucial indications for future studies, neurorehabilitation interventions, and clinical practice.

## Introduction

Each year about 500,000 people suffer a stroke. Strokes are the third leading cause of death in Western countries (after cardiovascular and neoplastic diseases) and one of the leading causes of long-term severe disability (Sudlow and Warlow, 1997; Pendlebury et al., 2009; Zhang et al., 2012). Indeed, it is a catastrophic and often unexpected event with a wide range of physical and psychological consequences that involve both patients and their relatives (Wolfe, 2000; Di Carlo, 2009). Due to the debilitating initial symptoms and long-term impairment in daily life activities like locomotion and speech, the consequences of a stroke depend on type, severity, and location of the occlusion. After a stroke, it is commonly possible to identify two basic categories of impairment or disability: motor disability (including the inability to walk, problems with coordination and balance, hemiparesis, or hemiplegia) and cognitive impairments (including aphasia, memory, and visuo-spatial and executive functions impairments) (Hendricks et al., 2002; Pohjasvaara et al., 2002; Hackett et al., 2005; Langhorne et al., 2009; Lloyd-Jones et al., 2009; Sundar and Adwani, 2010). The most common cognitive impairment after a stroke, which appears in approximately 50% of patients, is Unilateral Spatial Neglect (USN) (Bowen et al., 1999; Appelros et al., 2002; Nijboer et al., 2013a). USN commonly (in 90% of cases) occurs after lesions in the right hemisphere, particularly in the parietal (inferior), temporal (superior), and/or frontal (ventral) cortex and sometimes in subcortical nuclei (Buxbaum et al., 2004). This complex syndrome can be defined as “a failure to report, respond, or orient to contralateral stimuli that is not caused by an elemental sensorimotor deficit” (Heilman et al., 1985). Patients with USN may show several symptoms in everyday life, such as eating food only on the right side of the plate, putting make-up only on the right side of their face and, forgetting to look left before crossing the street (Nijboer et al., 2013b, 2014b). For these reasons, USN is a poor prognostic sign for both motor and cognitive rehabilitation outcomes (Buxbaum et al., 2004; Jehkonen et al., 2006; Mutai et al., 2012; Nijboer et al., 2014a).

An increasing number of theories have been proposed to explain the behaviors characteristic to USN; to date, the most interesting theories are attentional-based (Bartolomeo and Chokron, 2002; Corbetta et al., 2005; Corbetta and Shulman, 2011). Specifically, Bartolomeo said that “left neglect does not reflect an attentional deficit but an attentional bias consisting of enhanced attention to the right” (Bartolomeo and Chokron, 2002, p. 221). Indeed, Bartolomeo and Chokron argue that USN may be caused by an impairment in the exogenous (i.e., stimulus-related) orienting of attention because the endogenous (i.e., strategy-driven) way is relatively well-preserved, although it operates slowly (Bartolomeo and Chokron, 2002). In the same direction, Corbetta and colleagues (Corbetta et al., 2005; Corbetta and Shulman, 2011) argued that the attentional deficits in UNS may be mediated by a dysfunction, both functional and structural, of the two frontoparietal attention networks, in addition to damages resulting from the lesion (Corbetta et al., 2005; Corbetta and Shulman, 2011).

Paper-and-pencil tests are traditionally used to assess the presence of USN symptoms in a clinical setting. In “cancelation tasks,” patients are required to find a target symbol mixed with several other distractors. The most common tests are cancelation of line (Albert, 1973), letter (Diller and Weinberg, 1976), circle (Vallar and Perani, 1986), and star (Wilson et al., 1987). However, as noted by Rengachary et al. (2009), these paper-and-pencil tests may be particularly poor at detecting USN symptoms, especially in the chronic stage (Halligan et al., 1989). Driven by attentional-based theories, it is crucial to acknowledge that patients may be able to learn a compensatory attentional strategy and, consequently, to pass a test in which they have unlimited time to identify static targets. In clinical practice, two major methods for USN rehabilitation are visual searching and stimulation techniques: the first one is meant to improve voluntary exploration of the contralesional space (Pierce and Buxbaum, 2002; Paci et al., 2010), while the second one implicitly forces the patients to explore contralesional space (i.e., prismatic adaptation or caloric, galvanic, and optokinetic stimulation) (Kerkhoff and Schenk, 2012).

None of these approaches alone is the gold standard for rehabilitation of UNS (Pierce and Buxbaum, 2002; Bowen et al., 2013); it is strictly recommended that a combination of multiple approaches be used to develop a personalized rehabilitation process (Kerkhoff and Schenk, 2012).

Computerized methods offer a promising alternative approach for USN assessment and rehabilitation (Gontkovsky et al., 2002; Pflugshaupt et al., 2004; Deouell et al., 2005; Yong Joo et al., 2010; Bonato, 2012; Rabuffetti et al., 2012; Bonato and Deouell, 2013; Smit et al., 2013; Dalmaijer et al., 2014; Vaes et al., 2015). Computerized tests are able to identify subtle deficits that a static paper-and-pencil test might miss. Moreover, the traditional methods may lack ecological validity (which is crucial for rehabilitation) (Perez-Garcia et al., 1998; Levick, 2010), and there is often no correspondence between performance at the task and performance in real life (Eslinger et al., 1992, 2004; Vriezen et al., 2001). Finally, these protocols are time-consuming and tedious both for therapists and patients because people suffering from UNS also often experience anosognosia, meaning that they are unaware of their disability.

One of the most promising solutions to improve the quality of neuropsychological assessment and rehabilitation is the use of Virtual Reality (VR). VR can make more neuropsychological practice more involving, generalizable, and ecological thanks to its ability to measure behavior in valid, safe, and controlled environments objectively and automatically; dynamic learning also may increase engagement of the patients (Rizzo et al., 2002; Brooks and Rose, 2003; Riva, 2009; Sugarman et al., 2011). First, a systematic review about the potentiality of VR for USN assessment and rehabilitation was carried out by Tsirlin et al. (2009). They underlined that VR provides an advanced human-computer interface that allows the patients to interact with, and become immersed in, a computer-generated environment similar to the real-life experience. Thanks to this advanced technology, patients can be evaluated and trained through simulations that are relevant for everyday life, eliminating the necessity to use real environments that are not always available inside a hospital. VR can also improve traditional assessment methods by providing information about head and eye movements, postural deviations, and limb kinematics, which can be useful in detecting subtle deficits. Finally, Tsirlin et al. (2009) argued that VR assessment and rehabilitation of USN could be more engaging and consequently more effective than traditional methods. Despite the incredible potential of VR for assessment and rehabilitation of USN, Tsirlin et al. (2009) noted that there are several challenges that may limit future applications in this field: the ergonomic aspects of VR systems (considering the reduced mobility of post-stroke patients), the necessary collaboration between clinicians and technicians to set up VR systems, and the costs related to the design, maintenance and use of a VR system.

Thanks to the dramatic development of VR technology, several researchers have exploited the potential of VR both for the cognitive evaluation and rehabilitation of USN. On this basis, the main goal of this systematic review is to provide an overview of the latest applications in the field of assessment and rehabilitation of USN with VR applications since 2010 to provide crucial indications for future studies and neurorehabilitation interventions. Below, we analyze the articles and describe the methodology and technology used in the articles in order to understand the developments and new perspectives.

## Methods

We followed the Preferred Reporting Items for Systematic Reviews and Meta-Analysis (PRISMA) guidelines (Moher et al., 2009).

### Search strategy

To achieve this, a computer-based search in several databases was performed for relevant publications. Databases used for the search were: PsycINFO, Web of Science (Web of Knowledge), PubMed and Medline.

**Figure 1:**
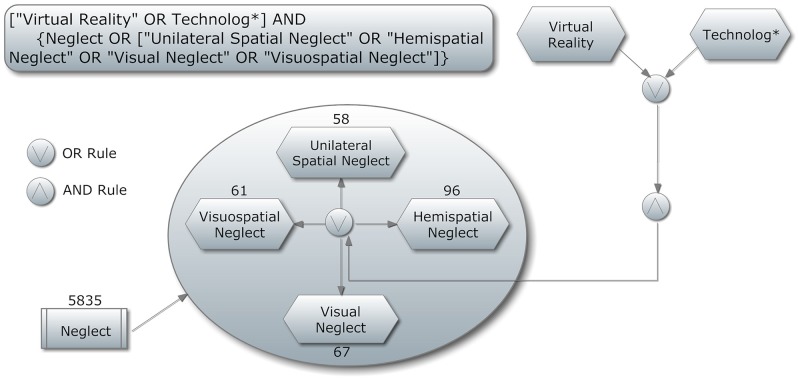
**Search strategy**. A graphical representation.

The search string was: (“Virtual Reality” OR Technolog^*^) AND [“Neglect” OR (“Unilateral Spatial Neglect” OR “Hemispatial Neglect” OR “Visual Neglect” OR “Visuospatial Neglect”)]. A graphical representation of the search string can been seen in Figure 1.

Our choice to search for both “virtual reality” and “technolog^*^” was to avoid missing papers due to the misleading terminologies that are often used in some studies. Acting within this strategy, we can be confident that this review is both replicable and inclusive of all possible records.

The articles were individually scanned to elaborate whether they fulfill the following inclusion criteria: (a) research article; (b) providing information about the used sample; (c) providing information about measures, and (d) published in English. These inclusion criteria were used for several reasons. As noted above, information about the sample and measures are a prerequisite.

The second search strategy (with the term Technolog^*^) had as a further exclusion criterion being present in the first list (already screened).

### Systematic review flow

**Figure 2:**
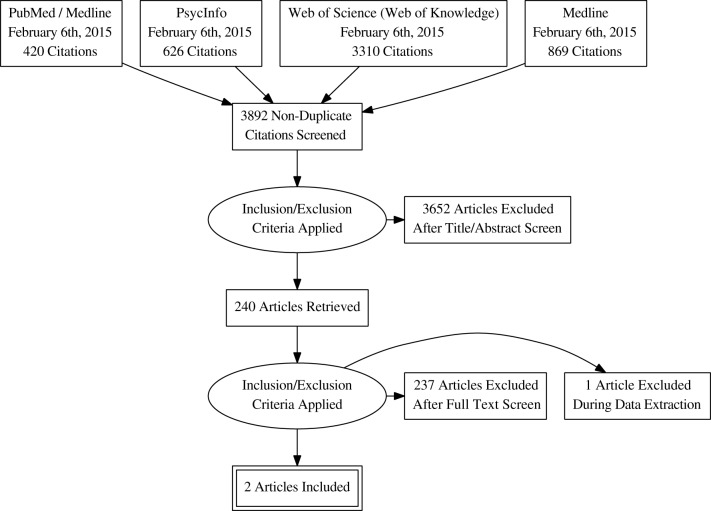
**Search strategy using “Virtual Reality” term**.

**Figure 3:**
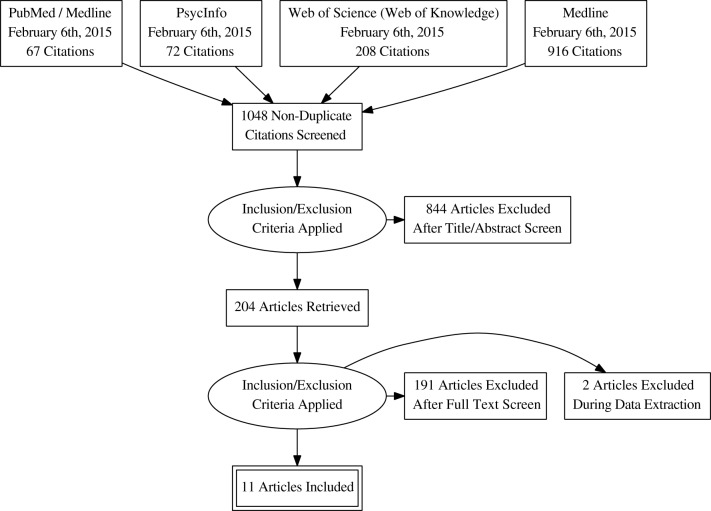
Search strategy using “Technolog^*^” term.

The flow chart of the systematic review is shown in Figure 2 for the term “Virtual Reality” and in Figure 3 for the term Technolog^*^. By searching in PsycINFO, PubMed, Medline and Web of Science (Web of Knowledge: WoK), our initial search yielded 1048 non-duplicate citations screened with “Virtual Reality” and 3892 with “Technolog^*^.” More details are available in the Search Strategy Table (Table 1). After the application of the inclusion criteria, papers were reduced to 204 and 240 articles, respectively. A deeper investigation of the full papers resulted in the exclusion of 191 and 237 articles, respectively. During the data extraction procedure, three additional full papers were excluded. In the end, 13 studies met the full criteria and were included in this review (Table 1). A flow diagram showing the procedure is detailed in Figure 2 for “Virtual Reality” search strategy and in Figure 3 for “Technolog^*^.”

**Table 1:** **Search strategy results**.

**“Virtual Reality” and**	**“Neglect”**	**“Unilateral spatial neglect”**	**“Hemispatial neglect”**	**“Visual neglect”**	**“Visuospatial neglect”**	**Other source**	**Total**
PsycINFO	52	5	7	4	4		72
Web of Science (Web of Knowledge)	112	18	35	25	18		208
PubMed	42	7	9	4	5		67
Medline	881	7	14	8	6		916
TOTAL	1087	37	65	41	33	12	1275
Non duplicated	911	13	57	31	24	12	1048
Excluded (after reading Title and Abstract)							844
Retrieved							204
Excluded (after applying inclusion criteria)							191
Excluded (missing experimental data)							2
Included							11
Technolog^*^ **and**	**“Neglect”**	**“Unilateral Spatial Neglect”**	**“Hemispatial Neglect”**	**“Visual Neglect”**	**“Visuospatial Neglect”**	**Other source**	**Total**
PsycINFO	591	7	11	12	5		626
Web of Science (Web of Knowledge)	3281	6	11	6	6		3310
PubMed	396	7	6	8	3		420
Medline	838	4	5	3	19		869
TOTAL	5106	24	33	29	33	12	5237
Non duplicated	3813	11	17	14	25	12	3892
Excluded (after reading Title and Abstract)							3652
Retrieved							240
Excluded (after applying inclusion criteria)							237
Excluded (missing experimental data)							1
Included							2

Expert colleagues in the field were contacted for suggestions on further studies to consider in our search. Four new studies arose and have been included in the analyzed studies. To assess a risk of bias, PRISMA recommendations for systematic literature analysis have been strictly followed. Three authors (E.P., S.S., and P.C.) independently selected paper abstracts and titles and analyzed the full papers that met the inclusion criteria, resolving disagreements through consensus.

## Results

In the current systematic review, we aim to provide a review of state-of-the-art experimental studies (from 2010 to 2014) focused on the use of VR for the assessment and rehabilitation of USN. In total, 12 studies met the inclusion criteria, were critically reviewed, and are summarized in Table 2.

**Table 2:** **Studies which met the inclusion criteria**.

**References**	**Characteristics of sample**	**Characteristics of VR applications**	**Sessions**	**Main outcomes**
**THE APPLICATION OF VR IN THE ASSESSMENT**				
Kim et al., 2010	Thirty-two post-stroke patients divided into two groups: .with USN (*n* = 16: 6 female, 10 male; mean age = 52.9, SD = 16.8; mean onset (months) = 3.9, SD = 3.2), .without USN (*n* = 16: 5 female, 11 male; mean age = 60.1, SD = 12.1; mean onset (days) = 2.2, SD = 1.7)	3D immersive VR program for street crossing. Patients had 16 missions: four missions at four different velocities. If patients failed to recognize the car approaching, they had visual and auditory cues to stop the avatar before failing their mission	Patients completed the two conventional neuropsychological paper-and-pencil tests (Line Cancellation Test and Line Bisection Test) on the same day they completed the 3D virtual street assessment	Deviation angle, left-to-right reaction time ratio, left visual auditory cue rates and left failure rate in the VR program showed significant differences between the two groups. Depending on the direction of approach of the virtual car, the left parameters were significantly higher than the right parameters in the USN group. Risky behavioral aspects in unilateral neglect patients can be safely detected using this program
Mesa-Gresa et al., 2011	Twenty-five post-stroke patients: 11 female, 14 male; mean age = 51.2, SD = 12.6; mean onset (days) = 504.4, SD = 335.1	The VRSCT (VR Street Crossing Test): during the training session, patients did a single task without traffic or other distractors. In the assessment session, the task consisted of twice crossing a two-way road to arrive at a supermarket and return. The task ended when patients went to and came back from the supermarket twice, making a maximum of four accidents	The cognitive assessment (BIT, CT ad CPT-II) was conducted during the same week as the virtual training. The training session took approximately 10 min and the evaluation session lasted until the patient finished the task and/or the patient was considered to have failed the task	Validity of VRSCT for the assessment of both negligent and non-negligent ABI patients VRSCT system correlated with BIT score for non-negligent patients. Negligent patients show more accidents than other patients. Also assessed the appropriate emotional response
Peskine et al., 2011	Nine post-stroke patients: 5 with USN and 4 without USN (4 female, 5 male; mean age = 50, SD = 15; mean onset (months) = 16.1, SD = 30.2. Nine control participants: 4 female, 5 male; mean age = 50.6, SD = 16.1;	Patients had to move in the city, locate a main target (swings in a park), and count all the bus stops. The town had 13 bus stops, six on one side and seven on the other side of the street	Neglect was assessed with the Bell test and the CBS. All subjects and controls received one session of virtual navigation	The main finding is that four patients who did not display USN on the cancelation task test, or in some cases on the behavioral scale, showed neglect symptoms on the virtual task
Buxbaum et al., 2012	Seventy post-stroke patients: 31 female, 39 male; mean age = 59.5, SD = 10.6; mean onset (months) = 29, SD = 23.7, 10 control participants: 5 female, 5 male; mean age = 62.2, SD = 15.1;	The VRLAT requires participants to travel along a virtual, non-branching path, either propelling themselves using a computer joystick (participant condition) or passively viewing the environment while an examiner navigates the path at a constant rate (examiner condition). Participants were asked to identify virtual objects on either side of the path and to avoid colliding with the objects	All participants completed a testing protocol (VRLAT and a real-world navigation task, tests of sensory and motor function, modified Bell Cancellation Test, Letter Cancellation and Line Bisection Tests, modified Fluff Test, laser Line-Bisection Task, and RWN) in approximately 90 min	The VRLAT demonstrated strong sensitivity and specificity, minimal practice effects, and strong validity, and outperformed traditional paper-and-pencil tests in the prediction of real-world collisions
Aravind and Lamontagne, 2014	Twelve post-stroke participants with USN: 8 female, 4 male; mean age = 60.7, SD = 8.6; mean onset (months) = 13.5, SD = 24.3	VR environment consisted of a room with a blue circular target on the wall at the far end and three red cylinders (the obstacles). In the locomotor obstacle avoidance task patient had to walking toward a target and avoid a collision with an moving object	The locomotor obstacle avoidance task, the tests for the diagnosis of USN (MVPT, and Star Cancellation), the clinical assessment (Bells Test, Line Bisection Tests, MOCA, and Trail Making Test-B), and hand dominance were administered on 2 separate days within 1 week	8 out of 12 participants collided with either contralesional or head-on obstacles or both. Delay in detection (perceptuo-motor task) and execution of avoidance strategies, and smaller distances from obstacles (locomotor task), were observed for colliders compared to non-colliders
Aravind et al., 2015	Twelve post-stroke participants with USN: 8 female, 4 male; mean age = 60.7, SD = 8.6; mean onset (months) = 13.5, SD = 24.3	VR environment consisted of a room with a blue circular target on the wall at the far end and three red cylinders, the obstacles. During the “obstacle detection task” one object approaching to the patients from the center, right or the left side of the room. When patient perceived the object had to push the button. During the “joystick-driven obstacle avoidance task” the patient is passively moved toward a target and must avoid objects that move at him. The patient may avoid the object moving to the right or left or up or slow down the speed of movement with the joystick	The obstacle detection task, joystick-driven obstacle avoidance task, the tests for the diagnosis of USN (MVPT, and Star Cancellation), the clinical assessment (Bells Test, Line Bisection Tests, MOCA, and Trail Making Test-B), and hand dominance were administered on 2 separate days within 1 week	In the detection task, the contralesional and head-on obstacles were detected at closer proximities compared to the ipsilesional obstacle. For the avoidance task, collisions were observed only for the contralesional and head-on obstacle approaches. For the contralesional obstacle approach, participants initiated their avoidance strategies at smaller distances from the obstacle and maintained smaller minimum distances from the obstacles. The distance at detection showed a negative association with the distance at the onset of avoidance strategy for all three obstacle approaches
Fordell et al., 2011	Thirty-one post-stroke patients divided into two groups:.with USN (*n* = 9: 3 female, 6 male; mean age = 73.3, SD = 12; mean onset = 2 weeks), .without USN (*n* = 22: 6 female, 16 male; mean age = 74.4, SD = 10.8; mean onset = 2 weeks)	VR-DiSTRO: virtual star cancelation, line bisection, visual extinction, Baking tray task. The patients used a robotic pen and shutter glasses for stereoscopic vision	The virtual and the classic versions of the test were administered with no time limits. Mean assessment time was 15 min for the VR-DiSTRO	VR-DiSTRO total score showed a 100% sensitivity and 82% specificity in accurately identifying USN patients
**THE APPLICATION OF VR IN THE REHABILITATION**				
Kim et al., 2011	Twenty-four post-stroke patients with USN divided into two groups: virtual reality (VR) group (*n* = 12: 3 female, 9 male; mean age = 62.3, SD = 10.2; mean onset (months) = 22.8, SD = 7.6) and the control group (*n* = 12: 7 female, 5 male, mean age = 67.2, SD = 13.9; mean onset (months) = 25.5, SD = 18.5)	The VR group received VR training with a system equipped with a monitor, a video camera and computer-recognizing gloves. There are three tasks: “Bird and Ball” (i.e., they had to touch a flying ball to turn it into a bird), “Coconut” (i.e., they had to catch coconuts falling from a tree) and “Container” (i.e., they had to move a box from one side to another). The control group received conventional neglect therapy such as visual scanning training	30 min a day, 5 days per week for 3 weeks. Both groups were assessed, before and after the training, with: Star Cancellation Test and the Line Bisection Test, CBS, and K-MBI	The changes in star cancelation test results and CBS in the VR group were significantly higher than those of the control group after treatment
Navarro et al., 2013	Thirty-two post-stroke patients divided into three groups:.with USN (*n* = 17: 5 female, 12 male; mean age = 58.5, SD = 10.1; mean onset (days) = 322.6, SD = 243.9), .without USN (*n* = 15: 7 female, 8 male; mean age = 50.8, SD = 13.5; mean onset (days) = 482.9, SD = 216.8). control group (*n* = 15: 3 female, 12 male; mean age = 54.6, SD = 5.7)	The VRSCT (VR Street Crossing Test): during the training session, patients did a single task without traffic or other distractors. In the assessment session, the task consisted of twice crossing a two-way road to arrive at a supermarket and return. The task ended when patients went to and came back from the supermarket twice, making a maximum of four accidents	One session divided into two parts: training (patients became acclimated to the hardware and software) and assessment (two consecutive repetitions of virtual street crossing). The neuropsychological assessment (BIT, CPT-II, Stroop Test, Color Trail Test, BADS—Zoo Map Test and Key Search Test) was made 3 days before or after the VR session	Patients with USN have a lack of efficacy in the task. That is, stroke subjects with USN received poorer results (higher values) than patients without USN, and stroke subjects as a whole received poorer results than healthy subjects
Mainetti et al., 2013	One right-hemisphere stroke patient with USN: Male, 65 years old, right fronto-temporal intraparenchymal hemorrhagic lesion in 2009	The “Duckneglect” platform, which included specially-designed games that require patients to reach targets with an increasing level of difficulties and visual and auditory cues	The rehabilitation lasted for half an hour each day, 5 days a week, for 1 month. with a follow-up 5 months later. A complete neuropsychological assessment (Line Cancellation Test, Letter Cancellation Test, Line Bisection Test, MMSE, Attentional Matrices and the Token Test).was done before, after and 5 months later the training	Significant improvement in the follow-up test, and a generalization to everyday life activities
van Kessel et al., 2013	Twenty-nine post-stroke patients divided into two groups:.control (*n* = 15: 5 female, 10 male; mean age = 59.1, SD = 6.8; mean onset (days) = 157.6, SD = 117.2), .experimental (*n* = 14: 7 female, 7 male; mean age = 61.8, SD = 7.8; mean onset (days) = 140.6, SD = 133.6)	New computerized training based on the “Visual Scanning Training” (TSVS) + Driving simulator tasks: in the first, they have to maintain their position in the middle of a street while an car moved at 50 km/h (Line Tracking Task); in the second, patients were asked to select a large rectangular dot target overlapping with the driving scene (Single Detection Task—CVRT); the third one was the combination of the previous two tasks	All patients received 30 training sessions (5 days a week, 1 h each day, for 6 weeks). A neuropsychological assessment (Line Cancellation Test, Letter Cancellation Test, Line Bisection Test, Bells Test, Word Reading Task, Gray Scales, and Baking Tray Task).was done before and after the training	No significant group and interaction effects were found that might reflect additional positive effects of dual task training
**INTEGRATED PLATFORMS**				
Tanaka et al., 2010	Two right-hemisphere stroke patients with USN: Patient A (female, 78 years old, parietal and temporal lobe infarction, onset 1 week) and Patient B (male, 62 years old, infarction in the middle cerebral artery territory, onset 49 weeks)	Using a head-mounted display (HDM), they administered different versions of the Line Cancellation Test: zoomed, normal or reduced, object-centered or with egocentric coordinates, with or without arrows	One session. Also the paper-and-pencil version of the Line Cancellation Test was administrated	The assessment of USN using an HMD system may clarify the left neglect area, which cannot be easily observed in the clinical evaluation for USN
Sugarman et al., 2011	One right-hemisphere stroke patient with USN: Female, 66-year old, massive right hemisphere stroke, onset 15 months	SeeMe system. Participants stood in a specific area in front of a large monitor that displayed the virtual scenes, seeing herself on the screen in real time, and being able to use trunk and limb movements to interact with the virtual environment	8 weekly 1-h treatment sessions using the SeeMe system. Three of the SeeMe tasks/games were used for treatment and a fourth task was used for evaluation. She was assessed on the first and last days of treatment	The right hippocampus plays a critical role in allocentric navigation, particularly when cognitive impairment is present

In the following paragraphs, we critically reviewed the selected studies by dividing them according to the main purposes of the virtual tools proposed: (1) neuropsychological assessment of USN symptoms; (2) neuropsychological rehabilitation of USN symptoms; and (3) comprehensive platform for both assessment and rehabilitation of USN symptoms.

### The application of VR in the assessment of USN

As it was described in the introduction, USN is typically evaluated by paper-and-pencil tests despite the aforementioned limitations of these tools. In this section, to deeply review the potential of VR for improving and/or integrating the traditional evaluations of USN, we analyzed the selected articles to provide an overview of the most recent virtual diagnostic tasks.

The first article analyzed was written by Kim et al. (2010), who used a 3D immersive VR program for street-crossing to assess USN in post-stroke patients. They assessed 32 patients, 16 with USN and 16 without USN. USN was assess by physiatrists and occupational therapists. They observe patients in the real life situations in order to find evidence of USN.

Patients was assesses during one session both with virtual and paper-and-pencil test. The test used are the Line Bisection Test (Schenkenberg et al., 1980) and the Line Cancellation Test (Albert, 1973).

At the beginning of virtual task, the patient see an avatar in front of a traffic light, the mission is cross the street without accident. If a car approaching to the avatar, patient have to push a stop button in order to avoid an accident. If patients failed to recognize the car approaching, they had visual and auditory cues to stop the avatar before failing their mission.

The results demonstrated that the two groups (patients with USN vs. patients without USN) showed differences in several variables analyzed during the task: deviation angle, left-to-right reaction time ratio, left visual, auditory cue rates, and left failure rate. Kim et al. (2010) showed that USN can be detected and measured easily and safely using their VR test. The authors also compared these virtual tools to the paper-and-pencil tests and found one correlation: the Line Bisection Test (Schenkenberg et al., 1980) correlated significantly with the deviation angle in the USN group.

In a similar test developed by Mesa-Gresa et al. (2011), they used a conventional LCD monitor, a surround system, a navigation and interaction joystick, and an optical tracking system (TRACKIR). Head movements were detected thanks to a cap with three reflecting markers and a USB infrared camera. A sample of 25 patients was analyzed, divided into neglect patients (*n* = 5) and non-neglect patients (*n* = 20) according to results obtained at the following tests: Behavioral Inattention Test (BIT), Color Trail Making Test (CTT), and Conners' Continuous Performance Test-II (CPT-II) (Peña-Casanova et al., 2006).

They planned a training session before the task that consisted of crossing a two-way road twice to arrive at a supermarket and then return. The task ended when patients went to and came back from the supermarket twice, making a maximum of four collisions with a car. They evaluated the following: how many times the participants looked to the left and to the right, the total time needed, the total number of accidents, whether the task was successfully accomplished, and a neuropsychological battery. During the VRSCT, negligent subjects showed a higher number of collision with a car than the other group, indeed indicating that the tool was able to discriminate between the two groups in clinical practice.

Peskine et al. (2011) developed a task that took place in a virtual city: patients have to count the number of bus stops they see. The sample included nine patients with a history of right cerebrovascular accidents (five of whom had visuospatial USN) and matched controls both for age and sex. USN was assessed using the Bells Cancellation Test (Gauthier et al., 1989) and the Catherine Bergego Scale (CBS; Azouvi et al., 2006). Patients used an HMD with an electromagnetic sensor system able to detect movements and sat on a swivel chair to turn on their own vertical axis. They had to move in the city, locate the swings in a park, and count all the bus stops; the examiner noted the patient's progress. The virtual assessment was done in just one session. Results showed that patients omitted more targets than controls and, most importantly, four patients without USN during the cancelation test showed USN in the virtual task.

Another navigation task was developed by Buxbaum et al. (2012). They created a “Virtual Reality Lateralized Attention Test” (VRLAT), a computerized measure of USN. They compared 71 USN patients with 10 control subjects. For the clinical assessment Buxbaum et al. (2012) used: a modified version of Bell Cancellation Test (Gauthier et al., 1989), the Letter Cancellation and Line Bisection Tests (Wilson et al., 1987), a modified version of the “fluff” test (Cocchini et al., 2001), a laser line-bisection task (Buxbaum et al., 2004), and a modified version of the Moss Real World Navigation (RWN) test (Buxbaum et al., 2008). During the VRLAT patient had to name all stationary objects in the scene while following a virtual winding path (i.e., navigation can be executed sometimes by the participants and sometimes by the experimenter). The program included three array conditions (i.e., simple, complex, and enhanced), and all patients completed all levels twice, once “coming” and once “going.” The software ran on a personal computer with a flat-screen video display; patients used a Logitech Attack 3 joystick.

This test seems to be better than traditional tests at predicting performance in real world. For this reason the VRLAT is a good tool for the assessment of USN. It's quick and easy to use, doesn't require specialized equipment, and could be useful both in clinical settings and in rehabilitation.

Aravind and colleagues (Aravind and Lamontagne, 2014; Aravind et al., 2015) developed a navigation task in a virtual room divided into three sub-tasks and analyzed the performance of 12 patients. A diagnosis of USN was based on the motor free visual perceptual test (MVPT; Colarusso and Hammill, 1972), and/or the Star Cancellation Test (Wilson et al., 1987). Clinical assessment included: Bells Cancellation Test (Gauthier et al., 1989), Line Bisection Tests (Wilson et al., 1987), the Montreal Cognitive Assessment (MOCA; Nasreddine et al., 2005), and the Trail Making Test-B (Army Individual Test Battery, 1944). Two of these tasks (“obstacle detection task” and the “joystick-driven obstacle avoidance task”) were analyzed in the first selected paper (Aravind et al., 2015); the other task, “locomotor obstacle avoidance task,” was described in another publication (Aravind and Lamontagne, 2014). Patients wearing a Visor SX60 head-mounted display (HMD) (NVIS, USA) and had a joystick (Attack3, Logitech, USA) to interact with the environment.

In the “locomotor obstacle avoidance task” (Aravind and Lamontagne, 2014) patient had to walking toward a target and avoid a collision with an moving object. The moving obstacle may approaching from center, right, or left.

During the “obstacle detection task” (Aravind et al., 2015) the patient was seated at a table with a joystick in the non-paretic hand. One of the three objects placed in center, right or left in the other side of the virtual room may approach toward the patient. When patient perceived the object had to push the button.

During the “joystick-driven obstacle avoidance task” (Aravind et al., 2015) the patient is passively moved toward a target and must avoid objects that move at him. The patient may avoid the object moving to the right or left or up or slow down the speed of movement with the joystick.

In the first task, patients detected contralesional obstacles at closer proximities compared to ipsilesional ones. For the “joystick-driven obstacle avoidance task,” participants begin to avoid objects at the last moment before the collision. Instead, they found that the performances on these paper-and-pencil tests were negatively associated with distances at detection, but the association lost significance with the exclusion of one patient (an outlier). For the “locomotor obstacle avoidance task,” Aravind and colleagues (Aravind and Lamontagne, 2014; Aravind et al., 2015) showed that 8 out of 12 subjects collided with either contralesional or head-on obstacles or both. Delay in detection and execution of avoidance strategies and smaller distances from obstacles were observed for colliders subjects compared to non-colliders one. After analyzing all three tasks, Aravind and colleagues (Aravind and Lamontagne, 2014; Aravind et al., 2015) argued that their system showed a typical pattern for USN patients and thus can be used for assessment.

The last article in this section is that of Fordell et al. (2011). They designed a VR Diagnostic Test Battery (VR-DiSTRO). The battery included the virtual version of four classical sub-tests: Star Cancellation and Line Bisection Test (Wilson et al., 1987), Visual Extinction Test (Geeraerts et al., 2005), and Baking Tray Task (Tham, 1996). During the experiment, patients have to do both virtual and classic versions of the test. The patients used a robotic pen (Phantom Omni haptic device) and shutter glasses for stereoscopic vision. All virtual tests took 15 min. The sample was composed of 31 post-stroke patients: 12 had a left-sided lesion and 19 had a right-sided one. VR-DiSTRO correctly identified the USN patients in the group, showing a 100% sensitivity and 82% specificity to correctly identify USN in the sample. Additionally, 77% of the sample said that the system was easy to use. The agreement with paper-and-pencil tests was moderate to almost perfect, indicating that this virtual battery was able to detect USN at least as well as the classic tests.

### The application of VR in the rehabilitation of USN

In order to investigate the potential of VR in USN rehabilitation, we provided an overview of the most recent studies showing different and alternative solutions compared with the traditional methods of rehabilitation. First of all, neuropsychological rehabilitation of USN must take into account the specific needs of each patient. For this reason, a more customizable neuropsychological application is essential.

The traditional rehabilitation methods are often characterized by repetitive exercises, non-consideration of the individual patients' differences and needs, and the inability to generalize the performance and outcomes as not measured and quantified. For instance, the prisms technique, one of the most effective techniques in the neuropsychological rehabilitation of USN, induces an optical shift of the visual field to the right; the patients have an adaptation to this visual distortion that reduces neglect symptoms (Rossetti et al., 1998; Jacquin-Courtois et al., 2013; Leigh et al., 2015). Between the various techniques it is the most effective one, but not yet to be widely used in clinical practice. For this reason there is a need for innovative rehabilitations methods able to decrease USN behavior for long-term.

Kim et al. (2011) examined 24 stroke patients with USN divided into two groups. The VR group (*n* = 12) received a VR training with a system equipped with a monitor, a video camera, and computer-recognizing gloves. Patients had to complete three tasks: “Bird and Ball” (i.e., they had to touch a flying ball to turn it into a bird), “Coconut” (i.e., they had to catch coconuts falling from a tree), and “Container” (i.e., they had to move a box from one side to another). The control group (*n* = 12) received conventional USN therapy such as reading, visual tracking, writing, drawing and copying, and puzzles. Both groups had daily sessions of 30 min day, five sessions per week for 3 weeks. Both groups were assessed with conventional USN tests such as: the Star Cancellation Test and the Line Bisection Test (Wilson et al., 1987), the CBS (Azouvi et al., 2003) and the Korean version of the Modified Bartel Index (K-MBI; Jung et al., 2007). Results showed that only the VR group improved in the Star Cancellation Test (Wilson et al., 1987) and in the CBS (Azouvi et al., 2003) after the rehabilitation period.

Navarro et al. (2013) assessed the clinical validation, usability, and convergent validity of the “Virtual Street Crossing System” (Mesa-Gresa et al., 2011) to find out if it could be used for rehabilitation of USN. Their sample was composed of 17 USN patients, 15 non-USN patients and 15 control subjects. The rehabilitation task was the same used by Mesa-Gresa and colleagues in their study (Mesa-Gresa et al., 2011) and described previously. After the virtual task patients were administered a modified version of the Short Feedback Questionnaire (SFQm; Witmer and Singer, 1998). Patients were also assessed with some neuropsychological tests like: BIT, CPT-II, Stroop Test, Color Trail Test, BADS—Zoo Map Test, and Key Search Test (Peña-Casanova et al., 2006). The assessment was administered 3 days before or after the VR session. Patients with USN showed a lack of efficacy in the task, for example, they made more accidents than other groups. The results of their study showed the clinical effectiveness of the street-crossing system as confirmed by the VR outcomes, and the correlation with the scores of the neuropsychological tests.

The “Duckneglect” platform was developed by Mainetti et al. (2013). They analyzed a single case in order to check the improvement in USN using their system for rehabilitation. The patient, IB, was a 65-year-old male with a right fronto-temporal intraparenchymal hemorrhagic lesion that occurred in 2009; he's right-handed and has had 18 years of education. This system included specially-designed games requiring patients to reach some targets through different levels of difficulty using visual and auditory cues. A webcam, connected to the host PC, was positioned frontally to the patient's face, and two loud speakers were positioned near the patient to create a spatialized sound. Video of the patient was acquired from the camera and real-time processed to extract his silhouette from the background. The silhouette was then pasted onto the virtual scene of the rehabilitation task. In the end, the final scene was displayed on a screen in front of the patient. Before and after rehabilitation training a fully neuropsychological battery was administered: Line Cancellation Test (Albert, 1973), Letter Cancellation Test (Diller and Weinberg, 1976), Line Bisection Test (Schenkenberg et al., 1980), the Mini Mental State Examination (MMSE; Folstein et al., 1983), the Attentional Matrices (Spinnler and Tognoni, 1987), and the Token Test (DE RENZI and Vignolo, 1962). The rehabilitation lasted for half an hour every day, 5 days a week for 1 month. 5 months later, the patient came back for a follow-up and exhibited a significant improvement both on a classic paper-and-pencil test and other neuropsychological tasks. The improvement was also present for activities of daily living.

van Kessel et al. (2013) analyzed the performance of 29 post-stroke (right hemisphere) patients during their rehabilitation with a new computerized training method based on the “Visual Scanning Training” (TSVS) of Pizzamiglio (Pizzamiglio, 1990). Patients were divided into two groups: the experimental group (*n* = 14) received the computerized training while control group (*n* = 15) received traditional training. All patients received 30 training sessions 5 days a week for 6 weeks, 1 h per day. They used several tests for pre- and post-training assessment: paper-and-pencil tests, observation scales and the Driving Simulator Tasks. The paper-and-pencil tests are: Line Cancellation Test (Albert, 1973), Letter Cancellation Test (Diller and Weinberg, 1976), Bells Cancellation Test (Gauthier et al., 1989), Line Bisection Test (Schenkenberg et al., 1980), Word Reading Task (Làdavas et al., 1997), Gray Scales (Tant et al., 2002), and Baking Tray Task (Tham and Tegnér, 1996). The observation scales include: Semi-structured scale for the evaluation of personal and extrapersonal neglect (Zoccolotti et al., 1992), and Subjective Neglect Questionnaire (Towle and Lincoln, 1991). In the Driving Simulator Tasks, patients had to perform three tasks: Line Tracking Task, Single Detection Task (CVRT), and a combination of the previous two tasks. During the training sessions, the TSVS was composed of the following exercises: Large Screen Digit Detection, copying lines drawn on a dot matrix, reading and copying training and figure description. During the first and third weeks, both groups received the same treatment: on Monday and on Wednesday they did the TSVS tasks and on Thursday and Friday they did the TSVS and the lane tracking. During the second and fourth weeks, patients worked for just 2 days: the experimental group did the TSVS and the dual task while the control group did the TSVS and the lane tracking. van Kessel et al. (2013) didn't find any significant group or interaction effects that might underline additional positive effects of the dual task training; they weren't the result of other factors like spontaneous recovery or learning effects.

### The application of integrated platform for USN

Two of the selected studies proposed integrated VR platforms that are useful both for assessment and rehabilitation of USN.

An interesting example was given by Tanaka et al. (2010), who developed an HMD for the assessment and rehabilitation of USN. They tested two post-stroke patients with USN using a combined system (Charge-Coupled Device camera, HMD, and a computer) programmed to show in the display a modified version of the classic Line Cancellation Test (Albert, 1973). They administered the standard paper-and-pencil task and six modified versions task created by manipulating the zoom (in or out), the coordinates of visual field (object-centered and egocentric), and the presence of cue (arrows). These manipulations have been made in order to find and identify the left neglect area. The study confirmed that, thanks to the special assessment through HDM, it was easier to identify the neglected area of the patients. These results might provide a more precise assessment and a more focused rehabilitation. Tanaka et al. (2010) showed that, with a reduced image condition and the arrows condition, performance at the cancelation task improved.

The other article was a feasibility study by Sugarman et al. (2011) proposing new tools that could be used both for assessment and rehabilitation: SeeMe. The system was tested on a single USN patient (66 years old) who had a right hemisphere stroke 15 months previously. The woman was invited to use the tool for 1 h each day for 8 weeks. The patient stood in a specific area in front of a large monitor that displayed the virtual scenes, seeing himself on the screen in real time and being able to use trunk and limb movements to interact with the virtual environment. A single screen-mounted camera and a vision-based tracking system captured and converted the user's movements. Three tasks were used for the rehabilitation and four for the assessment (i.e., React task, the patient have to touch the virtual balls that appear randomly on both sides of the screen). The patient was assessed on the first and on the last day of the treatment with SeeMe and with the standard paper-and-pencil tests. Also the SFQ (Witmer and Singer, 1998) an open ended interview was administered on the last day of treatment. To the SFQ (Witmer and Singer, 1998) patient assigns 5 points out of 5 in almost every question except the one that assesses whether the virtual environment looks real. To this question the patient assigns a score of 2 out of 5. For the assessment task results indicated a difference between movement times (defined as “the time elapsed between the appearance of the target and the subject's virtual contact with the target”) in the right and the left space. Moreover, after training there was an improvement in movement times for the neglected space and in the paper-and-pencil test for USN.

## Conclusions

The aim of this review is to describe and to critically analyze the most recent virtual tools developed and tested for the assessment and rehabilitation of USN in order to provide crucial indications for future studies, neurorehabilitation interventions, and clinical practice.

To date, traditional paper-and-pencil methods are still the most widely used technique in the clinical practice, despite several concerns both for assessment and rehabilitation of USN symptoms.

Regarding the assessment, the traditional paper-and-pencil tests may be deficient in detecting USN symptoms in the chronic stage of the disease (Rengachary et al., 2009), and their sensitivity and specificity varies between 38 and 52% (Agrell et al., 1997; Lindell et al., 2007; Fordell et al., 2011). On the other hand, regarding rehabilitative interventions, there is the prisms' technique, which is one of the most effective, but not the most used, techniques in neuropsychological rehabilitation of USN. It typically consists of sessions of repetitive exercises that have to be done several times a week but, unfortunately, have a limited effect in time (Rossetti et al., 1998; Newport and Schenk, 2012).

It is possible to note that paper-and-pencil tools use static, two-dimensional, and geometrical targets, which are far from those of a real, or virtual, environment. These tasks generally require a simple visual search in the near space, allowing only the diagnosis of peripersonal USN (Robertson and Halligan, 1999; Deouell et al., 2005; Kim et al., 2010; Aravind and Lamontagne, 2014). Otherwise, a real environment requires dynamic responses to the relevant stimuli that, in personal and extrapersonal space, change every time (Deouell et al., 2005; Buxbaum et al., 2008; Kim et al., 2010). This is a crucial feature of virtual environments since personal and extrapersonal USN are two subtypes of this syndrome that can be dissociated (Robertson and Halligan, 1999; Halligan et al., 2003). Specifically for rehabilitation, the use of moving stimuli may be crucial to modulate patients' visual attention; these kinds of objects can capture and drive attention to the left side of the space. Indeed, some recent evidence has reported that a moving cue in the left side of a task's space improved target detection in that area (Butter et al., 1990; Mattingley et al., 1994; Tanaka et al., 2010).

Moreover, both for static and moving stimuli there were different gradients of increasing reaction times, with a progression from the ipsilesional field toward the midline and into the contralesional field (Smania et al., 1998; Deouell et al., 2005; Dvorkin et al., 2007). Because of this feature, the computer version of reaction time tasks was generally more sensitive than paper-and-pencil tests (Rengachary et al., 2009; Bonato et al., 2012). One of the reasons for this behavioral pattern could be the predisposition of the patient with USN to initiating visual scanning of the environment from the ipsilesional side (Smania et al., 1998; Dvorkin et al., 2007; Aravind and Lamontagne, 2014; Aravind et al., 2015).

VR technologies offer impressive opportunities both for the rehabilitation and assessment of different cognitive deficits, including USN (Schultheis and Rizzo, 2001; Riva et al., 2004; Bohil et al., 2011).

According to the results of this systematic review, VR seems a promising instruments both for the assessment and rehabilitation of USN.

However, the trade-off between the incredible progress of VR and the need of methodological rigor and the possibility to the apply experimental protocols in the clinical practice has still to cope with different challenges.

First, as mentioned previously, Tsirlin and colleagues in their review (Tsirlin et al., 2009) underlined some characteristics of VR technologies that should be taken into consideration for future VR applications in this field.

The most important one is the ergonomic aspect of VR tools. Patients have specific needs to be considered, especially post-stroke patients who typically have to use a wheelchair for locomotion (Tsirlin et al., 2009). Our analysis showed that most of the selected studies have proposed VR assessment tools with greater attention paid to the ergonomic aspect in order to meet the needs of patients. In particular, it emerged that most of the recent VR systems could possibly be used with a chair or a wheelchair. Moreover, three selected studies have proposed some VR systems that can be easily controlled with one hand (Fordell et al., 2011; Kim et al., 2011), this is a great advantage for USN patients since hemiparesis is extremely common. Given this disability, it is very important to analyze usability aspects of the setting as Kim et al. (2011), Mainetti et al. (2013), Navarro et al. (2013), and van Kessel et al. (2013) did for their tools.

A second critical challenge for the clinician is the technical usability of the VR system/software since the clinical staff often has no programming skills. For this reason, cooperation with software developers is necessary for the use and customization of the technology. By designing intuitive VR applications and providing adequate training, developers may also help medical personnel in using these tools independently. First of all, Mainetti et al. (2013) emphasized the necessity of close collaboration between technical and clinical staff to tailor virtual environments to the specific requirements of patients. Moreover, three selected studies specifically addressed these issues, emphasizing the need for an easy-to-use application (Fordell et al., 2011; Sugarman et al., 2011; Sedda et al., 2013). Sugarman et al. (2011) have commented on their special attention to the usability aspects of their system, specifying that “SeeMe does not require any equipment beyond a webcam camera and a standard computer with a good video card” (p. 1). Indeed, there is a growing diffusion of VR-based telerehabilitation systems for post-stroke patients (for a review, see Brochard et al., 2010), which has allowed new directions for the design of ecological scenarios supporting multimodal interaction (Perez-Marcos et al., 2012).

The third important challenge that may limit the use of VR in the assessment and rehabilitation of USN is the high costs often required for designing and testing a technological system. Our analysis showed that only two selected studies have tried to pay particular attention to the costs (Kim et al., 2011; Mainetti et al., 2013), while the others tried to use cutting-edge technology in order to maximize the performance of the system.

Specifically for the neuropsychological rehabilitation of USN, it is essential to take into account the specific needs of the different patients. For this reason, a more customizable neuropsychological rehabilitation would be essential. A platform that allows the clinician to customize the tasks might also make a difference.

Finally, all the articles analyzed suggest several methods for the assessment and rehabilitation of USN, but there are some “methodological weaknesses.” Few studies compared VR methods with conventional ones (Kim et al., 2010, 2011; Mesa-Gresa et al., 2011; van Kessel et al., 2013; Aravind and Lamontagne, 2014; Aravind et al., 2015), only few studies compared the results with a control group (Kim et al., 2010; Peskine et al., 2011; Buxbaum et al., 2012; Navarro et al., 2013) and often the samples were too small to allow a generalization of the result (Peskine et al., 2011; Sugarman et al., 2011; Mainetti et al., 2013; Aravind and Lamontagne, 2014; Aravind et al., 2015), while controlled randomized trials testing the VR training in comparison with traditional protocol should be important. For further research, we also recommend adequate follow-up to maximize the benefits and monitor the persistence of the effect of neglect rehabilitation interventions. More, to enhance the potentiality of a multi-sensory and engaging VR stimulation, it is reasonable that USN patients should start a VR rehabilitation program in the acute stage.

However, the results obtained from the reviewed studies are promising and showed that VR systems stimulate interest and participation of patients (Kim et al., 2011). Indeed, VR simulations can be highly engaging by supporting a process known as “transformation of flow” (Riva et al., 2006), defined as an individual's ability to use and identify an optimal experience (i.e., flow) to promote new and unexpected psychological resources. This process may be particularly important since rehabilitation programs can be particularly demanding for patients. However, it is crucial to take into account potential transient side effects of immersive VR, such as cyber-sickness which occurs as a result of conflicts between visual, vestibular and proprioceptive signals. In addition to technological advancements, reducing the VR sessions (i.e., between 20 and 30 min) and giving precise explanations may alleviate any symptoms of discomfort.

Despite the great improvements in technology over the last 6 years, very few articles use new tools for assessment or rehabilitation in neuropsychology. The new technology systems for VR and the devices for “communication” with the virtual world could be very useful for neuropsychology; the possibility of acting in a virtual environment like in the real one is an important goal. Many efforts are aimed at improving the immersive virtual reality system, and there are two particularly important tools: VR wearable visors and the Cave Automatic Virtual Environment (CAVE). The VR head-mounted display is developed for virtual reality systems and video games. This tool uses custom tracking technology and creates a stereoscopic 3D view with excellent depth, scale and parallax by presenting unique and parallel images for each eye and using a 3-axis gyroscope, accelerometer and magnetometer to process data. The CAVE is a room with projection screens on the walls, floors and, in some cases, ceiling. The stereoscopic projectors are used for a 3D effect. These characteristics, together with the high-resolution of the graphics, allow an increase in the sense of presence. Users in the CAVE use head-trackers and hand-trackers in order to allow natural movements to interact with the virtual environment. CAVE is used mostly for design and fashion applications, but recently there have been some clinical applications, principally for the treatment of phobias and emotional disorders (Meyerbröker et al., 2010; Bouchard et al., 2013).

In terms of input devices, the classics are controllers for game consoles like Wii or Xbox. Wired gloves could be a way to improve the usability and comfort of the interaction and allow more fluid and natural movement in the environment. To remove the intermediation of tools, a solution could be using cameras to recognize models and identify motion, like Kinect or Vicon.

One input and output device is the Haptic device that allows people to feel the physical characteristics of the environment like gravity and viscosity.

The critical aspects of these devices are the high price and the complexity of both software and hardware components. To implement this device, support from technicians and developers is necessary in order to create the environments. Despite this limitation, this device has great potential to improve clinical practice. This new device allows for a completely different interaction with the virtual world and offers endless opportunities to analyze subject behavior in multiple ecological and controlled situations.

The aim of future studies could be to explore these possibilities in order to better understand the characteristics of each patient and his disorder and to create customized rehabilitation programs.

Additionally, the development of portable devices with good performance and reasonable prices may improve research concerning telemedicine, which would open the door to patients being treated at home without sacrificing medical supervision. Patients and doctors would be linked by a virtual platform that would allow monitoring of the patient's progress. The medical data could be mixed with the cognitive to make a complete picture of the healthy state of the patient. Medical data can be recorded by a wearable device capable of acquiring physiological signals.

### Conflict of interest statement

The authors declare that the research was conducted in the absence of any commercial or financial relationships that could be construed as a potential conflict of interest.
